# Complete genome sequences of *Escherichia coli* KA0011 clinical isolate used as a quality control strain of carbapenem susceptibility testing in Japan

**DOI:** 10.1128/mra.00155-24

**Published:** 2024-08-20

**Authors:** Ryohei Nomoto, Noriko Nakanishi, Shoko Komatsu, Mari Matsui, Satowa Suzuki

**Affiliations:** 1 Department of Infectious Diseases, Kobe Institute of Health, Kobe, Japan; 2 Antimicrobial Resistance Research Center, National Institute of Infectious Diseases, Tokyo, Japan; University of Maryland School of Medicine, Baltimore, Maryland, USA

**Keywords:** carbapenem-resistant Enterobacterales, genome analysis

## Abstract

*Escherichia coli* KA0011 had stable minimum inhibitory concentration values around the breakpoint range of meropenem and imipenem, making it suitable for use as a quality control strain for antimicrobial susceptibility testing. Here, we report the complete genomic sequence of KA0011.

## ANNOUNCEMENT

Carbapenem-resistant Enterobacterales (CRE) is critical in the World Health Organization’s priority pathogens list (1). Antimicrobial susceptibility test (AST) to determine the minimum inhibitory concentration (MIC) is crucial for evaluating CRE, but MIC results may vary by testing methods (2). Therefore, it is important to use reference strains with stable MIC values as quality control strains when conducting standardized AST.

We report the complete genomic DNA sequence of *Escherichia coli* ST131 strain KA0011 (O25:H4) from a peritonitis patient’s ascites, obtained through Japanese CRE case surveillance in 2018. The strain, with stable MIC values around the breakpoint range of meropenem and imipenem (1–2 µg/mL) in various ASTs and no carbapenemase production, is suitable for use as a quality control strain for AST with a lower risk of plasmid loss and is closer to the breakpoint concentration than the current Clinical and Laboratory Standards Institute quality control strains.

For genomic DNA extraction, *E. coli* strain KA0011, which was isolated from the ascites fluid of a patient with peritonitis, was cultured in tryptone soya broth at 37°C overnight. Genomic DNA was extracted using a NucleoSpin Tissue Kit (TaKaRa Bio, Shiga, Japan), according to the manufacturer’s protocol. The extracted genomic DNA was used for both Illumina and ONT sequencing. For Illumina sequencing, genomic libraries were prepared using the QIASeq FX DNA Library Kit (QIAGEN, Germany), and sequencing was performed using the Illumina MiSeq system with v.3 chemistry (2 × 300 bp). Default parameters were used for all software unless otherwise specified. Raw reads were quality-filtered and trimmed using fastp v.0.23.2 (3). Library preparation for Oxford Nanopore Technologies (ONT) sequencing followed the rapid barcoding DNA sequencing protocol with the SQK-RBK110.96 Kit (ONT, UK) without DNA shearing and size selection, and the libraries were sequenced using a single R9.4.1/FLO-MIN106 flow cell on a MinION sequencer Mk1B (ONT). Base calling was performed using Guppy v.6.4.2 in “high-accuracy” mode implemented on the MinKNOW software v.22.10.10 (ONT) (4). The ONT raw reads were demultiplexed, and ONT adapters were trimmed using Porechop v.0.2.4 (5). The number of reads is shown in Table 1.

**TABLE 1:** Assembly metrics and annotated features of *Escherichia coli* KA0011 strain

Sequence type	Genome size (bp)	No. of contigs	No. of Illumina reads	No. of nanopore reads	N_50_ of nanopore reads (bp)	DRA accession no.	Molecule type	Total length (bp)	Total no. of CDSs^*a*^	G + C content (%)	No. of rRNAs	No. of tRNAs	GenBank accession no.
131	5,207,510	4	1,539,360	30,392	17,526	DRR529737	Chromosome	5,118,800	4,807	50.7	22	90	AP029163
						DRR529738	Plasmid	81,994	102	51.6	0	0	AP029164
							Plasmid	5,167	6	47.5	0	0	AP029165
							Plasmid	1,549	2	51.0	0	0	AP029166

^*a*^ CDS, coding DNA sequence.

Hybrid assembly with ONT and Illumina data were performed using Unicycler pipeline v.0.4.8 (6) at the default settings previously described (7). The circularity of each contig was confirmed using the Unicycler log files. The chromosome and plasmid sequences were annotated using the DDBJ Fast Annotation and Submission Tool v.1.2.0 (8). Assembly metrics and annotated features are shown in Table 1.

The KA0011 strain contained three plasmids. From the determined complete genome sequence, the following antimicrobial resistance determinants were detected using ResFinder v.4.4.2 (9) (http://genepi.food.dtu.dk/resfinder) and AMRFinderPlus v.3.11.18 (10): *bla*_CTX-M-27_, *bla*_EC-5_-like, and *mdf*(*A*)*. bla*_CTX-M-27_ was found in two copies on a plasmid (size: 81,984 bp). Moreover, multiple chromosomal point mutations (ParC S80I, ParC E84V, ParE I529L, GyrA S83L, and GyrA D87N) that confer resistance to fluoroquinolones were found by ResFinder analysis.

## Data Availability

The complete genome sequences and raw sequence data of the four strains were deposited in DDBJ/EMBL/GenBank under the BioProject accession no. PRJDB17347. The accession numbers of the complete genome sequences are AP029163 (chromosome), AP029164 (plasmid 1), AP029165 (plasmid 2), and AP029166 (plasmid 3). The DRA accession numbers are DRR529737 (Illumina read data) and DRR529738 (ONT read data).
